# Molecular mechanism of Spinocerebellar Ataxia type 6: glutamine repeat disorder, channelopathy and transcriptional dysregulation. The multifaceted aspects of a single mutation

**DOI:** 10.3389/fncel.2015.00036

**Published:** 2015-02-16

**Authors:** Paola Giunti, Elide Mantuano, Marina Frontali, Liana Veneziano

**Affiliations:** ^1^Laboratory of Neurogenetics, Department of Molecular Neuroscience, UCL Institute of NeurologyLondon, UK; ^2^Laboratory of Neurogenetics, Institute of Translational Pharmacology, National Research Council of ItalyRome, Italy

**Keywords:** CACNA1A, P/Q type calcium channel, CaV2.1, Spinocerebellar Ataxia type 6, SCA6, polyglutamine disorder, channelopathy

## Abstract

Spinocerebellar Ataxia type 6 (SCA6) is an autosomal dominant neurodegenerative disease characterized by late onset, slowly progressive, mostly pure cerebellar ataxia. It is one of three allelic disorders associated to CACNA1A gene, coding for the Alpha1 A subunit of P/Q type calcium channel Cav2.1 expressed in the brain, particularly in the cerebellum. The other two disorders are Episodic Ataxia type 2 (EA2), and Familial Hemiplegic Migraine type 1 (FHM1). These disorders show distinct phenotypes that often overlap but have different pathogenic mechanisms. EA2 and FHM1 are due to mutations causing, respectively, a loss and a gain of channel function. SCA6, instead, is associated with short expansions of a polyglutamine stretch located in the cytoplasmic C-terminal tail of the protein. This domain has a relevant role in channel regulation, as well as in transcription regulation of other neuronal genes; thus the SCA6 CAG repeat expansion results in complex pathogenic molecular mechanisms reflecting the complex Cav2.1 C-terminus activity. We will provide a short review for an update on the SCA6 molecular mechanism.

## Introduction

Spinocerebellar Ataxia type 6 (SCA6, OMIM 183086) is a neurodegenerative disease characterized by late onset, slowly progressive, mostly pure cerebellar ataxia sometimes preceded by an episodic phase showing dysarthria, nystagmus and vertigo (Jodice et al., 1997). SCA6 is one of three autosomal dominant disorders due to mutations of CACNA1A gene. The gene encodes for the pore forming α1A subunit of P/Q type calcium channels Cav2.1, responsible for initiation of synaptic transmission at fast synapses (Catterall, 2000). Other auxiliary subunits cooperate in channel regulation (Catterall, 2000). SCA6 is due to small expansions of a CAG repeat stretch in exon 47, expressed only in some of the numerous isoforms of the CACNA1A gene as a polyglutamine sequence at protein level (Zhuchenko et al., 1997). The α1A subunit is a four domain-containing transmembrane protein of about 280 KDa with cytoplasmic N- and C-terminal regions. The cytoplasmic C-terminus, a 75 KDa polypeptide, contains residues involved in channel inactivation and modulation by intracellular signaling proteins (Catterall, 2000). The C-tail plays regulatory roles in the gating and trafficking of the channel, as reported also for the Cav1 family (Catterall, 2010). For Episodic Ataxia type 2 (EA2, OMIM 108500) and Familial Hemiplegic Migraine type 1 (FHM1, OMIM 141500) the molecular mechanism is clearly defined, a loss and a gain of channel function respectively (Guida et al., 2001; Tottene et al., 2002). In SCA6 the type of mutation (the polyglutamine expansion), the protein affected (the pore forming subunit of the P/Q type calcium channel) and the location of the mutation (the cytoplasmic C-terminal tail of the α1A subunit) suggest a more complex pathogenesis of the disease as opposed to a simpler gain of function model. Currently there is no treatment for SCA6, and understanding the underlying mechanism of the disease can be crucial in order to find molecular targets for therapeutic treatments. This review is focused on updating the recent advances in our understanding of molecular mechanisms of SCA6 pathogenesis.

## SCA6 as a polyglutamine disorder

SCA6 belongs to the group of autosomal dominant cerebellar ataxias (ADCAs) and, as with most ADCAs, the mutation is due to expansions of a polyglutamine repeat motif.

SCA6 shares features in common with other polyglutamine diseases, but differs in several aspects. The CACNA1A gene product is a membrane protein rather than a nuclear or cytoplasmic protein, as is the case in most polyglutamine disorders.

The formation of insoluble aggregates which leads to the production of intranuclear inclusions is the principal pathogenic feature in most of the polyglutamine diseases (Paulson, 1999). In SCA6, aggregates are rare, preferentially located in the cytoplasm as in SCA2 (Huynh et al., 2000; Ishiguro et al., 2010; Takahashi et al., 2013) and very rarely localizing to the nucleus in Purkinje cells (Kordasiewicz et al., 2006; Ishiguro et al., 2010). In SCA6 the normal repeat size ranges from 4 to 18 units (Zhuchenko et al., 1997) while that of the expanded alleles is from 20 to 33 repeats (Jodice et al., 1997; Yabe et al., 1998). Nineteen CAG repeats is considered an “intermediate allele”, predisposing to expansion into the abnormal range (Mariotti et al., 2001) or a susceptibility factor with variable penetrance and expression (Brenman, 2013). The pathological range falls within the distribution of normal alleles in other SCAs and below the threshold for polyglutamine aggregation, which typically is a number of units ranging from 35 to over 100 (Frontali et al., 1999). Furthermore, the small SCA6 repeat expansions are much more stable than in other polyglutamine disorders (Jodice et al., 1997; Zhuchenko et al., 1997).

A significant inverse correlation between age of disease onset and size of expanded alleles has been reported, as in other SCAs (Ishikawa et al., 1997; Zhuchenko et al., 1997; Maruyama et al., 2002), but an even closer correlation has been shown between the age of onset and the sum of CAG repeats in the normal and expanded alleles (Takahashi et al., 2004).

## SCA6 as a channelopathy

It is still unclear whether and how the SCA6 mutation exerts a pathological effect on the calcium channel function, aside from the weak toxic function of the polyglutamine expansion. Several studies attempting to determine the possible altered channel function reached highly variable, often conflicting results.

Matsuyama et al. (1999) studied the effect of 24, 30, or 40 polyglutamine expansions on channel properties in Baby Hamster Kidney (BHK) cells stably expressing α2δ and β1a auxiliary subunits, and first indicated that the 30–40 expanded polyglutamines directly alter channel function, causing a significant 8 mV hyperpolarizing shift in the voltage dependance of inactivation, which reduces the available channel population. This suggested that polyglutamine expansion in SCA6 leads to neuronal cell death and cerebellar atrophy through reduction in Ca^2+^ influx into Purkinje cells, while Ca^2+^ channels with 24 polyglutamines or fewer showed normal gating properties. Interestingly, Toru et al. (2000), in another study, detected a 6 mV hyperpolarizing shift in the voltage dependance of inactivation also using the 24 polyQ expanded allele, transfected into the HEK293 human cell expression system together with the human α2δ and β1a subunits. The crucial point of this experiment was that it has been performed using two splice variants of P/Q type Ca^2+^ channel exon 31+ and exon31− (Bourinet et al., 1999). The alternatively spliced CACNA1A gene exon 31 codes for two aminoacidic residues, asparagine and proline (NP) in the transmembrane domain IV. The resulting isoforms produce channels with distinct kinetics and generate P-type (NP−) and Q-type (NP+) channels expressed respectively in Purkinje and cerebellar granule cells. The negative shifts in voltage dependent inactivation were only observed in the variants of the P type Ca^2+^ channel NP−. These studies demonstrated that the effect of polyQ expansions on calcium influx results in reducing of Ca^2+^ entry into Purkinje cells (−NP isoform) and increasing Ca^2+^ entry into granule cells (+NP isoform). This provides an explanation for the selective Purkinje cell degeneration in SCA6. The hypothesis of SCA6 being a channelopathy was strongly supported by Restituito et al. (2000). They further elucidated the role of the different subtypes of the calcium channel subunits in determining the channel gating consequences of the mutation. In experiments performed in Xenopus oocytes, the SCA6 polyglutamine expansion shifted the voltage dependance of channel inactivation and the rate of inactivation only when expressed with the β4 subunit. In addition the mutation impairs the normal G-protein regulation of P/Q type Ca^2+^ channels, causing Purkinje cell degeneration through a possible gain-of-function mechanism with increased Ca^2+^ ion entry. Subsequently, studies in HEK293 cells stably expressing α2δ and β1 subunits showed that SCA6 Ca^2+^ channels do not have altered channel kinetics, but an increased current density attributable to a greater protein expression in the cellular membrane (Piedras-Rentería et al., 2001). In a follow up of these experiments, Chen and Piedras-Rentería (2007) obtained the same results using β2a or β4 auxiliary subunits, rather than the β1 subunit.

In summary, different model systems reached conflicting results. The isoforms of α1A subunit, the different auxiliary subunits and the cellular system seem to create the difference among the results obtained by different groups. On the other hand, studies performed on transgenic mice expressing polyQ expanded α1A subunit showed unchanged P/Q channel kinetics in cerebellar neurons (Saegusa et al., 2007; Watase et al., 2008).

## SCA6 as transcriptional dysregulation

Additional studies show a relevant role of the α1A subunit whole intracytoplamic C-terminal tail in SCA6 pathogenesis. Kordasiewicz et al. (2006) found that the C-terminal tail, from both the wild type and the mutant, is cleaved from the full-length protein and transported to the Purkinje cell nuclei equally. The SCA6 glutamine expansion is toxic to the cell only when inserted in its flanking sequence, indicating a mechanism for the pathogenesis of SCA6 which involves the whole C-terminal tail containing a nuclear localization signal and several protein binding sites. Despite the substantial evidence that the C-terminal fragment is conveyed to the nucleus, its potential role in this compartment was unknown. Interestingly, it has been reported that the C-terminus of the α1C subunit (Cav1.2, L-type calcium channel) is cleaved and is also present in cell nuclei where it acts as a transcription factor regulating a wide variety of endogenous genes (Gomez-Ospina et al., 2006). Recently Du et al. (2013) shed light on the origin and the function of this critical protein region. They demonstrated that a second cistron in the CACNA1A gene encodes a transcription factor, corresponding to the C-terminus, which coordinates the expression of neuronal genes involved in Purkinje cells development. They found a cryptic internal ribosomal entry site (IRES) located in a highly conserved sequence of 534 bp upstream the ATG 1960 (nucleotide 6,114 GenBank accession number NM_001127222) in CACNA1A mRNA, mediating the expression of the α1A C-Terminal (α1ACT) fragment. They further investigated the role of the wildtype and mutated α1ACT fragment in regulating gene expression. Through chromatin immunoprecipitation-based cloning experiments TAF1, BTG1, PMCA2 and GRN have been identified as target genes. These genes are abundantly expressed in Purkinje cell, although not uniquely, and they are possibly involved in the neurite outgrowth program. The wild type C-terminus increases the expression of at least these four genes, while the SCA6 mutated C-terminus abolishes this function and impairs the expression of the target genes in Purkinje cells, causing increased cell death and neurodegeneration. These results have been also obtained *in vivo*, in mice overexpressing α1ACT_SCA6_. These mice have reduced expression of TAF1, GRN, BTG1, PMCA2 genes and show ataxia and cerebellar cortical atrophy. On the contrary, overexpression of α1ACT_WT_ in α1A^−/−^ mice partially rescues their ataxic symptoms improving the phenotype at behavioral, histological and electrophysiological levels. Consistent with these findings, the TAF, BTG1, PMCA2 and GRN genes expression has increased from 1.5- to 3-fold.

## Conclusions

Currently, no therapy is known for SCA6, except for the use of Acetazolamide, a brain carbonic anhydrase inhibitor, that has been successfully used for EA2 but is possibly effective only in the episodic phase of SCA6 (Jen et al., 1998); however, Yabe et al. (2001) suggested that this drug can also temporarily reduce the severity of symptoms during the progression of the disease. Other therapies are in the experimental phase or their use is still controversial (Perlman, 2012) such as the NMDA antagonist (Ogawa et al., 2003; Ogawa, 2004) and branched-chain amino acids (BCAA), which improve neurotransmission among cerebellar neurons by stimulating intracellular glutamate metabolism (Mori et al., 2002). The latter treatment, used for other polyglutamine disorders, is likely to act on the effects of CAG repeat toxicity, in a later phase of the disease. In rare cases with Parkinsonism associated with SCA6, L-dopa has also been used (Khan et al., 2005). These different therapeutical approaches reflect the complex pathogenic mechanism of SCA6, which exhibits features of polyglutamine disease, channelopathy and dysregulation of transcription. The three proposed mechanisms seem to act according to divergent pathogenic pathways, but in some aspects they overlap and probably result in different symptoms occurring in different stages of the disease (see Table 1). The disease progression presumably occurs as the result of the synergy of the three mechanisms which lead to the selective Purkinje cells’ death and neurodegeneration.

**Table 1:** **Principal features of the SCA6 molecular mechanism**.

Polyglutamine disorder	Channelopathy	Transcriptional dysregulation
Formation of few insoluble protein aggregates mainly in the cytoplasm, very rarely in the nucleus.	*In vitro*: reduced Ca^2+^ influx into Purkinje cells and increased Ca^2+^ influx into granule cells depends on the involved isoform (NP+ or NP−).	The wild type and the SCA6 mutated Cav2.1 C-terminus localize in Purkinje cell nuclei equally.
The pathological range of the CAG repeat size falls within the normal range of the repeat size in other polyglutamine disorders.	*In vitro*: different subtypes of the calcium channel auxiliary subunits (β1, β2a and β4) determine different channel gating consequences of the mutation.	A second cistron in CACNA1A gene encodes a transcription factor corresponding to the C-terminus.
The small SCA6 repeat expansions are much more stable than other polyglutamine disorders	*In vivo*: transgenic mice expressing polyQ expanded α1A subunit show unchanged channel kinetics in cerebellar neurons.	The α1A C-terminus regulates the expression of genes involved in neurite outgrowth.
Inverse correlation between age of onset and size of expanded alleles. Closer inverse correlation between the age of onset and the sum of CAG repeats in the normal and expanded alleles.		*In vivo* the wild type C-terminus increases the expression of TAF1, GRN, BTG1 and PMCA2 genes in Purkinje cells. The mutated C-terminus abolishes this function.
Possibly responsible for the neurodegeneration	Possibly responsible for the early episodic symptoms	Responsible for the neurodegeneration

It is now clear that the Cav2.1 C-terminal tail has a relevant role in the multifaceted protein activity and that the SCA6 mutation alters most of the protein functions. It is to note that, besides the SCA6 polyglutamine expansion, the α1ACT harbors only mutations associated to EA2 and lacks FHM1 mutations. The properties of the wild type Cav2.1 C-terminal tail are similar to those shown by the analogous protein fragment of Cav1.2 of the L-type voltage-gated calcium channel, which also encodes a transcription factor (Gomez-Ospina et al., 2006). Cav2.1 C-terminus has a potential self-regulatory role, due to the presence of binding sites for proteins modulating channel activity, such as calmodulin, which interacts with most calcium channels (Dunlap, 2007). In contrast, EA2 mutations probably alter channel function, impairing the binding with other channel subunits or auxiliary proteins such as G proteins, SNARE proteins and CaMKII (Catterall, 2010).

Beyond binding sites for proteins regulating channel function, the C-terminus harbors AT-hook domains, corresponding to exon 44, which is present or spliced in different isoforms (Soong et al., 2002). This domain is a tripartite DNA binding motif that is specific for AT-rich sequences typically found in nuclear proteins belonging to the HMG (High Mobility Group), and DNA binding proteins (Aravind and Landsman, 1998). Moreover, the α1ACT harbors several binding sites for proteins regulating cleavage and translocation to the nucleus, where the cleaved fragment, or the α1ACT generated by IRES, exerts its transcriptional activity on genes involved in the neuronal phenotype, in neurogenesis or neurodegeneration. The α1ACT_SCA6_ abolishes the normal function of the wild type α1ACT, leading to a transcriptional dysregulation, as most of the other polyglutamine disorders (Ross, 2002). More studies are needed to fully understand the mechanisms underlying SCA6, which could possibly reveal pathways involved also in other neurodegenerative disorders and suggest therapeutical targets.

## Conflict of interest statement

The authors declare that the research was conducted in the absence of any commercial or financial relationships that could be construed as a potential conflict of interest.
